# Post-discharge Care Practices, Challenges, and Outcomes in Newborn Infants of Mothers With SARS-CoV-2 Infection: Insights From Public Hospitals

**DOI:** 10.7759/cureus.58734

**Published:** 2024-04-22

**Authors:** Uday P Patil, Arpit Gupta, Kevin Heringman, Cherbrale Hickman, Umesh Paudel, Elena V Wachtel

**Affiliations:** 1 Neonatal-Perinatal Medicine/Pediatrics, New York City (NYC) Health + Hospitals/Elmhurst and Icahn School of Medicine at Mount Sinai, New York, USA; 2 Neonatal-Perinatal Medicine/Pediatrics, New York City (NYC) Health + Hospitals/Metropolitan, New York, USA; 3 Pediatrics, New York City (NYC) Health + Hospitals/Elmhurst, New York, USA; 4 Neonatal-Perinatal Medicine/Pediatrics, New York City (NYC) Health + Hospitals/South Brooklyn Health, New York, USA; 5 Neonatal-Perinatal Medicine/Pediatrics, New York City (NYC) Health + Hospitals/Harlem, New York, USA; 6 Neonatal-Perinatal Medicine/Pediatrics, New York City (NYC) Health + Hospitals/Bellevue and New York University (NYU) Grossman School of Medicine, New York, USA

**Keywords:** care-utilization newborn, newborn covid, newborns of sars-cov-2 mothers, outcomes, homecare challenges, post-discharge care

## Abstract

Background

The data regarding the care at home and outcomes in infants of mothers infected with SARS-CoV-2 continue to evolve. There is a paucity of studies beyond the immediate newborn period. Our research aims to improve the understanding in these areas by studying the newborn population discharged from public hospitals in several boroughs of New York City (NYC) through the first year of the COVID-19 pandemic.

Objective

The objective of this study is to assess parental perspective and describe post-discharge care practices, patterns of healthcare utilization, challenges in obtaining care, and outcomes in infants between six and 12 months of age born to mothers infected with SARS-CoV-2 at the time of delivery.

Methods

We conducted an institutional review board (IRB)-approved multi-center retrospective cohort study of infants born to SARS-CoV-2-positive mothers at five NYC public hospitals between March and December of 2020. Clinical and demographic data were collected from electronic medical records. A phone interview of the caregivers using a standard questionnaire was conducted to collect data about care at home, healthcare utilization patterns, and challenges with access to healthcare.

Results

Our study cohort included 216 infants born to SARS-CoV-2-positive mothers with 16 (7.4%) mothers being symptomatic at discharge. Ten infants tested positive, and two showed symptoms before discharge. Two hundred seven (95.8%) infants were discharged home to their parents, and eight (3.7%) were transferred to other facilities. One hundred thirty-eight (66%) infants had at least one visit to the emergency room (ER) for various complaints where two were found to have COVID-19 with one needing hospitalization. One hundred seventy-two (79.6%) families responded to the phone interview. Most mothers (78%) cohabitated with their infants at home, and 70.3% elected to breastfeed. However, only 56.3% of mothers reported using all the recommended infection prevention practices at home. More than half (57%) of the families reported financial hardship related to the pandemic. Although 46.2% of patients missed their in-person health maintenance visits, telemedicine was highly utilized for follow-up with most being phone visits (70.3%). The majority of the infants (95.5%) remained up-to-date with their routine immunizations.

Conclusions

Our results suggest that infants born to SARS-CoV-2-infected mothers showed increased utilization of medical care and telemedicine between six and 12 months of age. Mothers reported low adherence to infection prevention practices at home; however, infants rarely showed clinically significant SARS-CoV-2 infection while maintaining high breastfeeding rates after discharge.

## Introduction

SARS-CoV-2 infection was first reported in late December 2019 from China with the World Health Organization (WHO) on March 11, 2020, declaring the novel coronavirus (COVID-19) outbreak a global pandemic. Cases rapidly emerged in other countries, and New York City (NYC) became an early epicenter of the COVID-19 pandemic in the United States with all patient groups including pregnant mothers and newborns getting affected [1-3]. Questions and concerns were validly raised regarding the transmissibility and the outcomes of COVID-19 in mothers and their newborns. The American Academy of Pediatrics (AAP) on April 2, 2020, provided the initial guidelines on the management of COVID-19 in exposed newborns with subsequent multiple revisions of these guidelines based on the evolving data [4]. A lot of those initial questions and concerns have been studied and reported widely since then by different authors, agencies, and institutions.

From these studies, we have learned that mothers with COVID-19 are at increased risk for premature labor and those with preexisting illnesses are at increased risk of morbidities such as the need for intensive care, mechanical ventilation, and extracorporeal support and death. Neonates born to mothers with COVID-19 are more likely to be premature and require intensive care, but the majority remain asymptomatic. The possibility of vertical transmission from a mother to her newborn seems less likely; however, this could be possible as postulated by some reports [5-9]. The horizontal transmission of COVID-19 to a newborn can occur during perinatal exposure, from infected family members or hospital staff while still admitted. Breastmilk and breastfeeding should be strongly encouraged for newborns as the chances of passing SARS-CoV-2 infection from the mother to the newborn causing serious illness are extremely low. Vaccinations for females who are pregnant, are planning to become pregnant, or were recently pregnant are safe and protect mothers from the serious consequences of COVID-19. Adequate (two doses) COVID-19 vaccination in mothers especially during the late second trimester and third trimester provides passive protective antibodies to newborns and reduces the overall severity of COVID-19 infection in newborns [5,9-12].

Some questions are yet to be adequately answered such as the outcomes of newborns and infants exposed to COVID-19 beyond the first few months of life, the impact of COVID-19 on post-discharge home care practices, and challenges, as well as the effect of COVID-19 on post-discharge healthcare utilization and outcomes. These answers are extremely important and should help us in the future with similar outbreaks. With NYC being one of the first and hardest-hit areas during the peak of the COVID-19 pandemic, it was very relevant for us to seek this information. Our study looks at the newborn population discharged from public hospitals in various boroughs of NYC through the COVID-19 pandemic. The objectives of this study were to evaluate the post-discharge care practices, patterns of healthcare utilization, challenges in obtaining care, and outcomes in SARS-CoV-2-exposed infants beyond six months of age while also assessing their parental perspectives.

This article was previously presented as a meeting abstract at the 2022 Pediatric Academic Societies Meeting on April 23, 2022.

## Materials and methods

We conducted a Biomedical Research Alliance of New York’s Institutional Review Board (BRANY-IRB)-approved multi-center observational study (approval number: 20-10-413-373) at five NYC public hospitals: NYC Health + Hospitals’ (NYC H + H) Elmhurst Hospital, Metropolitan Hospital, Coney Island Hospital, Harlem Hospital, and Bellevue Regional Perinatal Center. We included in our study a cohort of infants born to SARS-CoV-2-positive mothers at these hospitals during the first wave of the COVID-19 pandemic between March and December of 2020. Infants not born at these hospitals and those lost to follow-up were excluded. The study design had two components. The first was a retrospective chart review, in which clinical and demographic data on mothers and infants were collected from the electronic medical records. This included data regarding the mode of delivery, mother-infant contact after birth, breastfeeding while in the hospital, and disposition at the time of the infant’s discharge from the hospital. We included in the study all the infants born during the above time period and did not exclude infants based on their gestational age or the severity of illness or other criteria.

The second component of our study included telephonic interviews with the caregivers of the infants in the study cohort. For this, we used a standard questionnaire that was developed by our study team solely for the purpose of the study. The questionnaire consisted of a total of 25 questions (see Appendices) that included questions regarding demographic information and questions related to the care practices after discharge from the hospital. This questionnaire was administered after obtaining consent from the caregivers. The data about newborn care at home, breastfeeding practices, infection prevention methods used, post-discharge healthcare utilization patterns, challenges with access to healthcare, and the overall caregiver perspective were captured using this questionnaire. The infants in our study cohort were between six months and 12 months of chronological age at the time of the administration of the questionnaire to their caregivers.

Data were obtained from electronic medical records, and phone interviews were used. The data are confidential, transparent, and reliable. The information obtained is used exclusively for the purpose of this research study. Data were tabulated in Excel spreadsheets (Microsoft Corp., Redmond, WA) after de-identifying private information, and then, statistical analysis was performed using Statistical Package for Social Sciences (SPSS) version 23.0 (IBM SPSS Statistics, Armonk, NY). Descriptive statistics were used to report patient demographics and clinical characteristics. Continuous data are presented as means with standard deviation. Categorical data were analyzed using frequencies and shown in percentages.

## Results

Clinical characteristics and outcomes of the newborn infants born to mothers with COVID-19 while admitted to the hospital

Our study cohort included 216 infants born to COVID-19-positive mothers. The majority of these mothers were asymptomatic throughout their hospital stay. Sixteen (7.4%) mothers were symptomatic at the time of discharge. Contrary to the prevalent practices published during the early stages of the pandemic, the mode of delivery in our study was unaffected by the COVID-19-positive status of the mother with 143 (66.2%) mothers delivering vaginally [13]. Out of 216 mother-baby dyads, 138 (63.8%) had mother-baby contact by either early skin-to-skin care or direct breastfeeding before the testing of the newborn. This is also reflected in the high breastfeeding rate of 60.6% in our study population. Although the large majority of the newborns remained in their mother’s room (61.1%) during their hospital stay, only 10 infants (4.6%) tested positive, and two (0.9%) showed symptoms of respiratory distress needing the escalation of care with mechanical ventilation for pneumonia [9]. Two hundred seven (95.8%) infants were discharged home to their parents, while eight (3.7%) were transferred to another facility, and one newborn was sent to foster care (Table 1).

**Table 1:** Demographic and clinical characteristics of newborn infants of SARS-CoV-2-infected mothers SD, standard deviation; pCO_2_, partial pressure of carbon dioxide; pO_2_, partial pressure of oxygen; APGAR, appearance, pulse, grimace, activity, and respiration; NICU, neonatal intensive care unit; EBM, evidence-based medicine

Characteristics	N = 216
Gestational age in weeks, mean + SD	38.16 + 3.44
Birth weight in grams, mean + SD	3140 + 590.31
Time since the rupture of membranes in hours, mean + SD	4.52 + 6.35
Umbilical arterial cord blood gas pH, mean + SD	7.27 + 0.074
Umbilical arterial cord blood pCO_2_, mean + SD	50.56 + 11.66
Umbilical arterial cord blood pO_2_, mean + SD	26.86 + 11.95
Umbilical arterial cord base deficit, mean + SD	4.24 + 3.09
Mode of delivery	
Vaginal	143 (66.2)
Cesarean section	73 (33.8)
APGAR scores, median	
1 minute	9
5 minutes	9
Gender, n (%)	
Male	112 (51.9)
Female	104 (48.1)
Mother-baby contact before testing, n (%)	
No	78 (36.2)
Yes	138 (63.8)
Placement/location of the infant	
Mother’s room	132 (61.1)
NICU	68 (31.5)
Isolation for the baby only	15 (6.9)
Others	1 (0.5)
Newborn separated from the mother, n (%)	
No	110 (50.9)
Yes	106 (49.1)
Infant’s bed type during admission, n (%)	
Bassinet	71 (32.9)
Isolette	144 (66.7)
Others	1 (0.5)
Breastfeeding, n (%)	
No	85 (39.4)
Yes	131 (60.6)
EBM use during hospitalization, n (%)	
No	213 (98.6)
Yes	3 (1.4)
Discharge disposition, n (%)	
Home	207 (95.8)
Transfer	8 (3.7)
Others	1 (0.5)

Post-discharge healthcare utilization and outcomes

We assessed the follow-up care sought by the infants in our study cohort using their electronic medical records. In this cohort, 138 (66%) infants out of 209 had at least one visit to the emergency room (ER) for various complaints, whereas two (1.4%) were found to have COVID-19 with one needing hospitalization. Eighty-two out of 204 (40.1%) infants had at least one sick visit to our pediatric clinics for various reasons. We noticed a large number of missed in-person visits for routine infant health maintenance (46.2%); however, there was a large number of infants who received telehealth visits (73.8%), and a majority of the infants (95.5%) remained up-to-date with their routine immunizations. Of the 199 infants, nine (4.5%) infants missed routine immunizations, three (1.5%) infants showed developmental delays, and 10 (5%) infants had inadequate weight gain (Table 2).

**Table 2:** Post-discharge healthcare utilization and outcomes *Variable based on care utilization and/or loss to follow-up

Healthcare utilization	N*	N (%)
Infants with at least one emergency room visit post discharge, n (%)	209	138 (66)
Infants with more than one emergency room visit post discharge, n (%)	209	29 (13.8)
Infants with at least one sick visit to the pediatric clinic, n (%)	204	82 (40.1)
Infants with missed routine health maintenance visits, n (%)	199	92 (46.2)
Infants with missed immunizations, n (%)	199	09 (4.5)
Infants showing developmental delays, n (%)	199	03 (1.5)
Infants showing inadequate weight gain, n (%)	199	10 (5)
Infants receiving telehealth visit, n (%)		
Video visits	199	07 (3.5)
Phone-only visits	199	140 (70.3)

Post-discharge care practices and challenges

Study team members who were trained in conducting standardized interviews for this study reached out to the caregivers of the infants in our cohort via telephone calls. After obtaining their consent, a phone interview of the caregivers was carried out using a standard questionnaire that was developed for our study. A total of 172 (79.6%) caregivers responded to the interview. Among these, the majority of the mothers (78%) cohabitated with their infant in the same room at home with most (70.3%) electing to breastfeed with a mean duration of breastfeeding being about 25 weeks. However, only 56.3% of mothers reported using all the recommended infection prevention practices at home. Lastly, more than half (57%) of the families reported financial hardship such as loss of job or significant decrease in the salary related to the pandemic (Table 3).

**Table 3:** Post-discharge care practices and challenges SD: standard deviation

Care practices and challenges from parental interviews (N = 172)	
Median number of people in the household, N (range)	4 (1-11)
Mothers cohabitated with their infants, N (%)	134 (78)
Mothers reported direct breastfeeding at home for any duration, N (%)	121 (70.3)
Mean duration of breastfeeding at home in weeks, N (+SD)	25.14 (+17.7)
Mothers reported using expressed human milk at home, N (%)	117 (69.6)
Infection prevention practices by mothers at home, N (%)	
Hand washing only	6 (3.4)
Hand washing and mask use	65 (37.7)
Mask and gloves	1 (0.6)
All precautions	97 (56.3)
Financial hardship, N (%)	
Families reporting COVID-19-related job loss/financial difficulties	98 (57)

## Discussion

Our observational descriptive study uncovers important information that sheds light on not only the care at home but also the care sought by the families of infants born to mothers with COVID-19. Our study was conducted at five high-risk, public hospitals in NYC, which were battling an extraordinary surge of COVID-19 infections during the period of March 2020 to December 2020. During this time of the pandemic, the percentage of positive COVID-19 tests was at its peak (65%) [1]. All the participant hospitals in our study cared for immigrant, multicultural, ethnically diverse, and medically underserved populations. They were affected much before the American Academy of Pediatrics released its guidelines on the management of newborns exposed to SARS-CoV-2 [4]. In the setting of variations in international approaches and the paucity of standardized guidelines on infection prevention efforts after birth, each hospital in our study implemented its own procedures or protocols to prevent the transmission of SARS-CoV-2 to newborns while in the mother-baby unit or in the neonatal intensive care unit (NICU). These included the universal screening of parturient mothers for COVID-19, strict utilization of personal protective equipment (PPE) by caregivers and the healthcare staff, use of barriers such as curtains, and use of isolette for the placement of newborns. Each hospital made all the efforts to minimize the separation of the newborns from their mothers and to promote exclusive breastfeeding of the newborns [14,15]. This approach was different than the available information from the literature at that time [13]. These precautions in the hospital settings prevented the additional transmission of SARS-CoV-2 from mothers to newborns as evidenced by only 4.6% of newborns in our study cohort acquiring SARS-CoV-2 infection before hospital discharge. Moreover, among symptomatic mothers, only two infants were noted to have symptomatic COVID-19 during hospitalization. The low rate of symptoms and positive test results echoes the results of other studies of SARS-CoV-2-exposed infants during the early months of COVID-19 [2,5].

When investigating the impact of COVID-19 on routine healthcare and care utilization in our study cohort, we noticed that some of our findings were consistent with similar observational studies. In a cross-sectional survey of caregivers across the United States under 12 years of age, Teasdale et al. reported an overall rate of 41.3% of children missing their routine healthcare visits with approximately 25% under the age of two years missing their routine visits [16]. The primary reasons for missed visits were the fear of COVID-19 exposure, followed by the doctor’s office being closed. The missed vaccination rate in their study was 33%. The rate of missed routine care visits in our study was similar (46.2%) and could also be attributed to the increased fear of COVID-19 exposure in this vulnerable group. This rate is definitely higher than the standard no-show rate of 15%-30% in the pediatric population [17]. Interestingly, the combined rate of telemedicine visits (phone and video) in our study was high compared to that by Teasdale et al. [16] (73.8% versus 29.8%). It is also important to note that the missed vaccination rate in our group was far lower (4.5%) than the national average of 33.1% reported in the aforementioned study. We think that this could be attributed to the innovative approach taken by most of our ambulatory pediatric sites in deliberately scheduling health maintenance visits for infants in a dedicated timeslot or location during the pandemic. This may have alleviated the fear of the caregivers in our population. On the other hand, when looking at ER visits, our study showed an increased proportion of visits in infants compared to other studies that showed a drop in pediatric ER visits in the year 2020 compared to 2019 while acuity and admissions went up [18,19]. This increase in ER visits in our study is difficult to understand but could be because of the increase in parental anxiety during the peak of the pandemic coupled with the perceived reduced availability of regular clinic appointments. Although our study reported a very low proportion (1.5%) of the infants showing concerns of developmental delays, it is difficult to diagnose developmental delay at ages 6-12 months given the lack of completed development of major physiologic milestones. Alarmingly, recent studies have reported an increased risk of developmental delays in COVID-19-exposed newborns between 18 and 24 months of age and stressed the need for close follow-up of these newborns [20,21].

Our study has the unique advantages of being conducted in the most diverse population in New York City and being able to provide the assessment of the parental perspectives after discharge from the hospital. The females in our study are high-risk obstetric candidates, as well as at high risk of COVID-19 exposure and morbidity due to the sociodemographic makeup of our population [22,23]. The various mother-baby friendly measures taken during the pandemic helped us to maintain a high breastfeeding rate in the NYC H + H hospital system that continued after discharge from the hospital. Combined with low rates of newborn COVID-19 infection, our approach supports the AAP’s recommendations on the safety of breastfeeding while the mother is positive for COVID-19 infection while minimizing the negative effects of parents-baby separation [24,25]. Our study also demonstrates the usefulness of telemedicine in a pandemic setting for newborn infants. It is worth mentioning that the higher utilization of telemedicine may partly be attributable to the healthcare facilities’ preference toward telehealth during the pandemic. Nonetheless, we noted that the parents found it very useful and calming to meet their pediatrician through telemedicine. These findings support similar experiences reported in Europe [26]. High routine immunization rates were maintained, and ED visits remained average.

Our study did have limitations mainly due to the changing guidelines during the year of the COVID-19 pandemic such as the timing of testing, isolation of newborns from their mothers, and discharge planning for newborns of mothers with COVID-19 [27]. The hospitals in our study had to adapt to those guidelines, while the different boroughs of New York City involved had different peak infection rates and timelines. Recollection bias during the caregiver interviews and patients lost to follow-up may pose additional limitations. We did not survey the post-discharge outcomes for infants born to COVID-19-negative mothers to draw comparisons with our study population.

## Conclusions

Our results suggest that infants born to SARS-CoV-2-infected mothers showed increased utilization of medical care and telemedicine between six and 12 months of age. Although about half of the mothers reported adherence to full infection prevention practices at home, infants seldom showed clinically significant SARS-CoV-2 infection while maintaining high breastfeeding rates after discharge.

## Appendices

Sample of the phone interview questionnaire for caregivers

Demographic Questions

1. Do you consider yourself to be Hispanic or Latino/a?

Yes

No

Do not know

Refused

2. What race do you consider yourself to be? (clarify if needed, read options if necessary)

White or Caucasian

Black or African American

Hispanic or Latino

Asian (includes South and Southeast Asian)

Native Hawaiian or Pacific Islander

American Indian or Alaska Native

Others (record response)

Do not know

Refused

3. What is the highest grade (or year) of school you have completed? (do not read categories)

8th grade or less (elementary)

Grades 9-11 (some high school)

Grade 12 or general equivalency diploma (GED) (high school graduate)

College 1-3 years (some college or technical school)

College four years or more (college graduate)

Others

Do not know

Refused

4. How many children do you have, and how old are each of them?

5. How many people have been living in your household since you came home with your baby?

6. Do you or did you have any financial difficulties, lost jobs during the COVID-19 pandemic?

Care-Related Questions

1. Were you able to have your baby in your room after delivery while you were in the hospital?

Yes: all the time

No

Partially: for some time

2. Were other caregivers for the baby such as the father or grandparents or other family members of the baby allowed to visit?

Yes. If yes, who was allowed?

No

3. (To the mother) Where you able to breastfeed while in the hospital?

Yes. If yes, exclusive or with supplementation?

No

4. Where you able to provide expressed (pumped) breastmilk to your baby while in the hospital?

Yes

No

5. Who was able to pick up the baby from the hospital on the day of the baby’s discharge?

Father

Grandparent

Designated family member

Designated friend

6. (To the mother) Did you share the same room with your baby after coming home?

Yes

No

7. What precautions did you implement at home while caring for your baby?

Washing hands

Wearing a mask

Wearing gloves

8. (To the mother) Are you breastfeeding at home now?

Yes

No

9. If no, were you able to breastfeed for any period of time after discharge? If yes, for how long?

_____ weeks

_____ months

10. Were you able to provide expressed (pumped) breast milk to your baby after coming home from the hospital?

Yes

No

11. Did you allow visitors at home?

Yes. If yes, what precautions did you use for the visitors?

No

12. Has anyone at home been sick around you or your baby since you came home from the hospital?

Yes. If yes, did you consult the baby’s doctor or do COVID-19 testing?

No

13. Were you able to bring the baby for the follow-up appointments? If not you, who did?

Yes

No

If no: Father

          Grandparent

          Designated family member

          Designated friend

14. Were you provided with a tele-visitation option?

Yes. If yes, telephone or video visit?

No

15. Have you missed any follow-up appointments with the pediatrician?

Yes. If yes, what was the reason?

No

16. Are your baby’s vaccinations up-to-date?

Yes

No. If missed vaccinations, please specify

17. Has your baby needed any prescribed medications after discharge from the hospital? If so, what medications, and did you have any difficulties obtaining them from the pharmacy?

Yes. If yes, please write the names of the medications

No

18. Has your baby been to the emergency room after coming home as a newborn?

Yes. If yes, how many times, and for what reasons?

No

19. Has your baby been admitted to the hospital after coming home as a newborn?

Yes

If yes: What hospital/s?

            For how many times?

            For what reasons?

No
